# Recent Advances in Bioplastics: Application and Biodegradation

**DOI:** 10.3390/polym12040920

**Published:** 2020-04-15

**Authors:** Tanja Narancic, Federico Cerrone, Niall Beagan, Kevin E. O’Connor

**Affiliations:** 1 UCD Earth Institute and School of Biomolecular and Biomedical Science, University College Dublin, Belfield, 4, D04 N2E5 Dublin, Ireland; tanja.narancic@ucd.ie (T.N.); federico.cerrone@ucd.ie (F.C.); niall.beagan@ucdconnect.ie (N.B.); 2 BiOrbic - Bioeconomy Research Centre, University College Dublin, Belfield, 4, D04 N2E5 Dublin, Ireland; 3 School of Biomolecular and Biomedical Sciences, Earth Institute, O’Brien Centre for Science, University College Dublin, Belfield, 4, D04 N2E5 Dublin, Ireland

**Keywords:** biodegradable polymers, medical application, drug delivery systems (DDS), bioscaffold, biophotonics, packaging application, biodegradation

## Abstract

The success of oil-based plastics and the continued growth of production and utilisation can be attributed to their cost, durability, strength to weight ratio, and eight contributions to the ease of everyday life. However, their mainly single use, durability and recalcitrant nature have led to a substantial increase of plastics as a fraction of municipal solid waste. The need to substitute single use products that are not easy to collect has inspired a lot of research towards finding sustainable replacements for oil-based plastics. In addition, specific physicochemical, biological, and degradation properties of biodegradable polymers have made them attractive materials for biomedical applications. This review summarises the advances in drug delivery systems, specifically design of nanoparticles based on the biodegradable polymers. We also discuss the research performed in the area of biophotonics and challenges and opportunities brought by the design and application of biodegradable polymers in tissue engineering. We then discuss state-of-the-art research in the design and application of biodegradable polymers in packaging and emphasise the advances in smart packaging development. Finally, we provide an overview of the biodegradation of these polymers and composites in managed and unmanaged environments.

## 1. Introduction

Plastics are a large family of polymers, traditionally derived from fossil resources that are characterised as having a broad range of properties and characteristics [1]. Approximately 90% of plastics produced are derived from fossil feedstocks [2]. Currently, plastic production accounts for approximately 4–8% of oil consumption globally, and this is expected to reach 20% by 2050 [3,4]. Ever since their wide scale production in the 1950s, plastics have permeated society due to their use in a wide range of applications [5]. The success and continued growth of plastics can be attributed to their low prices, durability, strength to weight ratios, and contributions to the ease of everyday life [6]. With their low cost coupled with a wide range of properties, global plastic production has steadily increased from 15 million metric tonnes in 1964 to 359 million metric tonnes in 2018, with a projected 2-fold increase within the next 20 years [2,7,8]. Plastics have contributed greatly to the ease of everyday life as a result of their large-scale utility and their global presence as packaging materials for the collection and storage of drinks and food. The plastic industry is important for employment, given the reported over 1.6 million people employed in the industry across the EU, and the €360 billion turnover in 2018 [8]. Furthermore, the high strength to weight ratio of plastics allows us to reduce the transport costs of goods. For instance, the use of polyethylene terephthalate (PET) bottles over glass counterparts has been noted to reduce energy consumption associated with transport by 52% in Europe [2,6]. 

Global economic growth and the improvement in living standards has resulted in an increase in purchasing power by individuals, and so has contributed to the increase in plastic production. While traditional petrochemical plastic products have improved the quality of everyday life, their mainly single use, durable, and recalcitrant nature has led to a substantial increase as a fraction of municipal solid waste. Approximately 25.8 million tonnes of post-consumer plastic waste is generated in Europe annually, of which 30% is recycled, with a further 40% destined for incineration [9]. That still leaves the large volume of plastic waste posing a great environmental problem [2]. Currently, plastic packaging makes up approximately 40% of the plastic market, of which most is destined for single use [10]. In 2013, 72% of plastic packaging was not recovered for recycling, of which 40% ended up in landfill, and a further 32% leaked into the environment. Current statistics estimate that at least eight million tonnes of plastics leak into the ocean each year [2,4]. It has been noted that between 4.6 and 12.7 million tonnes of plastic waste produced by 192 coastal countries in 2010 was found in the ocean [10], with single use plastics making up half of marine pollutants across its beaches [11]. Current statistics released by The European Commission state that 1.5–4% of the global plastic produced leaks into the ocean annually [9,12]. Furthermore, approximately 79% of all plastic ever produced has not been recycled [5], generating a large volume of plastic waste. There are reports on the leakage of toxic chemicals, plastic debris, and microplastics that have had a devastating impact on coral reefs, marine mammals, and terrestrial life [13,14,15]. The leakage of phthalates used as plasticisers during the plastic manufacturing process into the environment has raised concerns over human and animal health, potentially increasing the risk of cancer in humans [16].

Landfilling has been widely employed for the end-of-life “management” of plastic waste. Not only does it require vast amounts of space, negative consequences related to the contamination of surrounding soils and ground due to by-products such as phalates and bisphenols associated with plastic physical deterioration leaking into the surrounding groundwater and soil have been reported [6,16]. Additionally, since none of the material is recovered, this waste “management” system is entirely linear and does not reduce virgin resource utilisation, stifling the vision of a circular economy [17]. Mechanical recycling of plastic is the most common recycling practice, and involves collection, washing, sorting based on plastic type, and grinding of the material into smaller fragments for remoulding. However, over time, the continued recycling of a plastic results in a deterioration in its desirable properties when compared to the virgin material. Currently, a poor recycling rate of approximately 14% for single use plastics can be observed globally [18]. Recycling rates vary widely across Europe, with many countries still employing landfilling as the first or second option for plastic waste treatment. However, countries such as Switzerland and Germany have implemented landfilling restrictions and have achieved landfilling rates of less than 10% [19]. In Europe, recycling recently overtook landfilling in post-consumer waste treatment in 2016 with a recycling rate of 31.3% and a landfilling rate of 27.3% [19].

While incineration reduces the requirement for landfilling and facilitates energy recovery, incineration of plastics must comply with environmental control measures imposed via the EU Hazardous Waste Incineration Directive; 2000/76/EC [20]. There are concerns that hazardous substances such as dioxins, furans, and polychlorinated biphenyls released into the atmosphere during the burning process pose a danger to the environment and human health [17,21]. The European Union, under the 7th Environmental Action plan, has outlined that all member states must end incineration of recyclable materials and reach a recycling rate of 50% by the year 2020 [22]. 

Since plastics pose a serious problem to terrestrial and aquatic environments, and current strategies for plastic waste reduction while showing progress still are not the best suited (incineration), or do not reach the set targets (recycling), we have to look at other materials as replacements for widely used plastics. Biobased and biodegradable plastics can contribute to a more sustainable society through the use of renewable resources and contribute to the reduction in CO_2_ emissions during production. Furthermore, biodegradable plastics offer new end-of-life management options, such as anaerobic digestion or composting [1], that have lower or no negative impacts on the environment. Our own research has spanned the synthesis of biodegradable polymers [23,24,25] the conversion of non-degradable polymers to biodegradable polymers [26,27,28], the application of polymers [29,30,31], and the biodegradation of such polymers [1,32,33]. We have attempted to bring this breadth of knowledge into a single review, contrarily to the many previous reports which treat the topics separately. This review article is a one-stop resource discussing recent advances in applications of bioplastics. It provides a brief overview of biodegradable plastics and their properties, followed by recent advances in their applications in medicine, with an emphasis on nanoparticle-based drug delivery systems, biophotonics and challenges and opportunities brought by the design and application of biodegradable polymers in tissue engineering. The following sections discuss the recent research efforts in design of packaging based on biodegradable polymers, and critically assesses the biodegradability of biodegradable polymers.

## 2. Bioplastics

**Figure 1:**
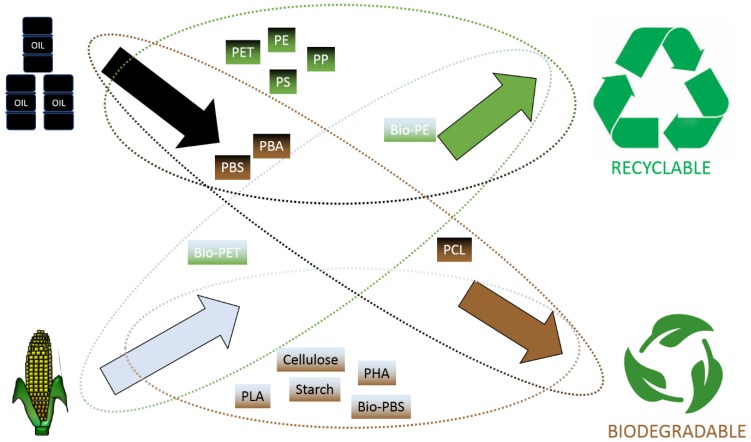
Four-way diagram of origin vs. end-of-life management for different polymers. Polymers originating from fossil fuels (polyethylene terephthalate (PET), polyethylene (PE), polystyrene (PS), polypropylene (PP)), or from renewable resource(s) (polybutylene succinate (PBS), polybutylene adipate (PBA), polycaprolactone (PCL)) can either be recycled or biodegraded. Biobased polyethylene terephthalate bio-PET and biobased polyethylene bio-PE are made from renewable resources and are recyclable. Polylactic acid, PLA; polyhydroxyalkanoates, PHA; biobased polybutylene succinate Bio-PBS are made from renewable resources and are biodegradable. *Adapted from Emadian et al. 2017 and Narancic et al. 2018* [1,35].

Currently, thermoplastics such as polyethylene terephthalate (PET), polyethylene (PE), polypropylene (PP), and polystyrene (PS) make up a total of 60% of the overall plastic demand in Europe [19]. While these plastics are traditionally petrochemically derived, there is a growing demand for the production of plastics using renewable resources (so called “bioplastics”) as alternatives to their petrochemically derived counterparts (Figure 1). All bioplastics are produced starting from natural resources. However, not all are biodegradable [10,34]. Bioplastics are a family of plastics that can be divided into two categories, biodegradable and non-biodegradable (Table 1). The diversity of biodegradable bioplastics is found in the variation in biodegradation rates and routes. Such plastics include polylactic acid (PLA), polyhydroxyalkanoates (PHA), cellulose, and starch. Similarly to oil-based plastics, biodegradable biobased plastics can be recycled or incinerated, but they are not widely recycled, as they are seen as contaminants in the current recycling system. They may also be microbially degraded, allowing for alternative end-of-life management, such as industrial and home composting, anaerobic digestion, depending on the plastics’ type, facilitating the development of a circular economy [1,35]. Non-biodegradable bioplastics include bio-polyethylene terephthalate (bio-PET), polyol-polyurethane, and bio-polyethylene (Bio-PE).

Oil-based plastics can also be categorised similarly based on their biodegradability (Figure 1). For instance, while PE and PS are not biodegradable, polycaprolactone and polybutylene adipate terephthalate (PBAT) are [34]. The production of plastics traditionally derived from petrochemical sources such as PET and PE, from natural resources, has garnered interest from plastic manufacturers, as the same processing equipment can be used in its manufacturing, thereby lowering total investment in infrastructure for their manufacture as the material properties are the same as conventional PET and PE resins [36]. The production of biobased PE from sugar cane in Brazil has been successful [37], while PET entirely derived from plant material for the production of soft drink bottles has recently been achieved (bio-PET) [38]. Even though these plastics are biobased and can contribute to reducing greenhouse emissions in plastic production through, for example, the growth of plant biomass which uses carbon dioxide, their bonding is identical to their petrochemical versions, and so they are not biodegradable [36]. Meanwhile, the industrial production of the biodegradable plastic, polybutylene succinate (PBS), (traditionally petrochemically derived) from sugarcane, cassava and corn (Bio-PBS) has been underway since 2017 [39] highlighting the move towards sustainable, biobased, biodegradable plastic alternatives.

While bioplastics remain a niche with only 1% of total plastic production, there is a movement towards their wider deployment. The global biodegradable plastic market is projected to reach $6.73 billion by 2025 from $3.02 billion in 2018 [41]. The main driver for this dramatic increase is due to the increasing demand for biodegradable polymers in emerging economies such as India, Brazil, and China [41]. While starch blends have the highest share in the biodegradable plastics production, polyhydroxyalkanoates (PHA) and polylactic acid (PLA) are reported to be the main contributors to the growth of biobased biodegradable plastics [42]. PHA and PLA have a market share of 1.2% and 13.9% of the bioplastic market respectively (% wt of 2.11 million tonnes of bioplastics produced). PHA is expected to see a 6.3-fold increase in global production from 25 320 tonnes 2019 to 159 700 tonnes by 2024 while PLA is expected to see a 8% increase production from 293 290 tonnes in 2019 to 317 000 in 2024 [43].

PHAs are biodegradable, optically active polymers synthesised by many bacteria as a stress response during inorganic nutrient deprivation, such as that of nitrogen, phosphate, or oxygen, while carbon is in excess [44,45,46,47]. PHA is produced via microbial fermentation processes and subsequently extracted by microbial cell lysis. PHAs are biocompatible, biodegradable, non-toxic polyesters consisting of (*R*)-3-hydroxyalkanoic acids [28] and exhibit similar thermoplastic properties to petrochemical plastics [46,48]. The variations in their physical properties are due to the diversity of their monomeric compositions, and thus, they cater for a wide array of applications. PHA is classified based on the length of the carbon chain of the PHA monomer: short chain length (*scl*) PHA consisting of 4 or 5 carbons in length and medium chain length (*mcl*) PHA consisting of 6–14 carbons in length [47,49,50,51]. The final properties of PHA, such as the degree of crystallinity, melting temperature (T_m_), and glass transition temperature (T_g_) are heavily dependent on the monomer composition of the polymer, which is influenced by the organism, the conditions of growth, and the method of polymer extraction. *scl*PHA typically display properties closest to conventional plastics like polypropylene, while *mcl*PHA displays more elastomeric properties [52,53]. Poly-3-hydroxybutyrate (PHB) is the most widely studied PHA polymer and exhibits brittle and highly crystalline characteristics similar to those of polypropylene [54]. However, blending of PHA monomers, creating co-polymers is often undertaken in order to tailor the thermal and mechanical properties of the polymer to the desired characteristics by varying the composition [55,56,57]. For instance, poly-3-hydroxybuterate-co-3-hydroxyvalerate (PHBV) is known to be more desirable than PHB homopolymer due to its lower melting temperature and lower percentage crystallinity, making it easier to mould and less brittle [58,59]. PHA’s current market share is very small. Only 25,200 tonnes were produced in 2019 accounting for 1.2% of the overall bioplastic market, a 1.7-fold decrease compared to the previous year [43]

Polylactic acid (PLA) is a biobased and biodegradable polyester representing 13.9% of global production capacities of bioplastic in 2019 [43]. The PLA monomers, L and/or D-lactic acid, are produced via microbial fermentation and further chemically polymerised to yield PLA. The final properties of PLA, the degree of crystallinity, melting temperature, and glass transition temperature are heavily dependent on the content of lactic acid enantiomers within PLA chains. PLA homopolymers containing either optically pure L-lactic acid or D-lactic acid are semi-crystalline polyesters with a melting temperature (T_m_) of about 175 °C and a glass transition temperature (T_g_) of around 55 °C, while PLA heteropolymers (poly DL-lactic acid) are amorphous due to disordered polymer chains [60], also with a T_g_ of around 55 °C [61].

Starch is a biodegradable, biobased polysaccharide consisting of amylose and amylopectin and is synthesised by most plants via photosynthesis [62]. Starch is an abundantly available, renewable, and cheap biopolymer, widely used for packaging in the food industry. Starch-based polymers represented 21.3% of global production capacities of bioplastic in 2019 [43]. However, naturally occurring starch is limited in its application, as its processability as a thermoplastic polymer is heavily affected by hydrophilicity, intermolecular forces, and hydrogen bonds present in the polymer, resulting in a high T_g_ and low T_m_ [63]. In order to overcome this, starch is further processed by mixing with plasticizers such as glycerol, urea, sorbitol, or glycerine, in the presence of elevated temperatures and shear forces, to facilitate the improvement in plasticity and thermoplastic characteristics of the polymer, yielding the thermoplastic polymer, thermoplastic starch (TPS) [63]. According to Zhang *et al.*, 2014 [64] most TPS possess a glass transition temperature (T_g_) within the range of −75 to 10 °C, and that varies depending on the TPS plant source and plasticiser employed; a melting temperature (T_m_) of 150 °C was recorded for TPS homopolymer [65]. TPS may be blended with biodegradable polymers such as PLA and PHA, and polycaprolactone or synthetic polymers such as polyethylene, polypropylene, and polystyrene in order to alleviate any shortcomings with the mechanical properties of a TPS homopolymer. TPS have applications as compost bags and in the food packaging industry and as films for seafood, meat, and vegetable industries. More in-depth reviews of the TPS biopolymer are available in the literature [62,63,64]. 

Cellulose is the most abundant natural biopolymer on earth sourced predominantly from trees and cotton [66], and is one of four constituents of plant cell walls [67]. Approximately 1.5 × 10^12^ tonnes is produced annually [68]. Cellulose is a linear homopolymer of 7000–15,000 β-D glucose monomers which alternately rotate 180° and form microfibrils with diameters of approximately 3–4 nm, and subsequently, macrofibrils of 10–25 nm in diameter [67,69]. Naturally occurring cellulose possesses a multi-level microstructure commonly referred to as a hierarchical structure. Nanocellulose particles maybe be isolated from Agri and forestry sourced cellulose by destroying the native hierarchical structure of cellulose via enzymatic, chemical, and/or physical methodologies which have been previously summarised [70]. Meanwhile, the microbial secretion of nanocellulose fibres has been previously reported in the literature [71,72] and is attracting increased interest in the medical industry due to its high purity and biocompatibility compared to cellulose sourced via plant biomass [71]. According to Mariano *et al*., 2017 [73], cellulose nanoparticles do not become soft at elevated temperatures, which represents a difficulty in measuring the polymer’s T_g_ and T_m_; however, the author states that possible values for the T_g_ and T_m_ of nanocellulose range from 220–250 to 430 °C respectively. The applications of nanocellulose include nanocellulose-based nanocomposites in the medical industry, water filtration, reinforcement of Li-ion battery manufacturing, and applications in the food packaging industry to name a few [73,74]. Furthermore, comprehensive reviews of nanocellulose biopolymers are available in the literature [69,70,72,75].

## 3. Medical Applications

Biodegradable polymers have been at the forefront of research for biomedical applications in the last 50 years. The advancements have been seen in the areas of using biodegradable polymers as delivery vehicles for controlled drug release [76,77,78] and development of therapeutic devices [77,78,79], including implants and three-dimensional scaffolds for tissue engineering [80,81,82,83].

### 3.1. Biodegradable Polymers as Drug Delivery Systems

#### 3.1.1. Nanoparticle Systems as Drug Nanocarriers

Biodegradable polymers as drug delivery systems (DDS) should be manufactured in a way that takes advantage of their potential for self-assembly into nanocarriers that can be loaded with specific drugs. These biodegradable polymeric DDS should fulfil two major requirements: performance and safety. In addition, the DDS have to be capable of maximum encapsulation efficiency of the target drug [84] have increased residence time in the target tissue, maximum bioavailability to achieve a therapeutic effect, and biodegradation in a timeframe compatible with the healing of the target tissue [85]. At the same time these biodegradable polymers should be safe; i.e., not inducing in vivo toxicity and not promoting an inflammatory response by the immunological system [86]. The physico-chemical features of the biodegradable polymer that dictate their success as nanocarriers are: polymeric composition, surface charge, molecular weight of the polymer, tacticity of the monomers, colloidal stability, size distribution of the nanoparticles, minimization of nanoparticle aggregation, and their hydrophilicity to hydrophobicity ratio [87]. Examples of efficient DDS typically take advantage of the amphiphilic nature of the nanocarrier and of the hydrophobic nature of the drug to deliver the therapeutic load tissue-specifically. 

#### 3.1.2. PHA-Based Nanomedicine for Potential DDS

**Figure 2:**
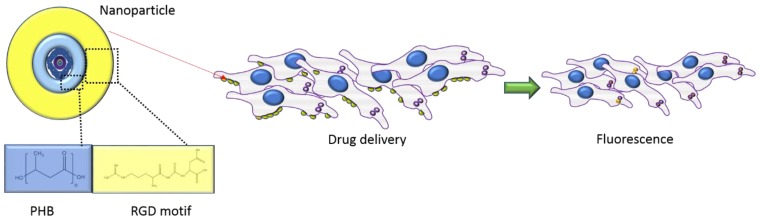
Polyhydroxybutyrate-Arg-Gly-Asp-oligopeptide (PHB-RGD)-based nanoparticles as a drug delivery system to deliver a fluorescent drug to cancer cells in vitro. *Freely adapted from Kim et al. 2009* [89].

PHA-based DDS were covered in an extensive review by Barouti et al., 2017 [88], wherein a synthetic approach in producing PHA was highlighted. Some specific examples of the use of PHA for DDS are briefly described here: Kim et al., 2009 [89] used a fusion polyhydroxyalkanoate synthase (PhaC) with an oligopeptidic RGD motif; that is recognized as extracellular matrix ligand in cancer cell adhesion, creating, effectively, PHB micelles able to self-assemble in an aqueous environment and that are taken up by the α/β integrin of the cancer cells that mediate the cellular adhesion (Figure 2). Specific uptake of these nanocarriers was visualized by a fluorescence microscope when a fluorescent dye was loaded into these micelles (Figure 2).

González-Miró et al., 2018 [90] produced PHB beads by using an inducible expression of a fusion protein composed by a PhaC and a modified, non-toxic pneumolysin equivalent of a virulence factor produced by *Streptococcus pneumoniae*. This system induced a better immunogenic response, and also increased a broad range immune response towards pneumolysin of different serotypical origin. Kim et al., 2014 [91] utilised polyhydroxyoctanoate/polyethylene glycol (PHO/PEG) copolymer nanoparticles to load paclitaxel (an anticancer drug) by nanoprecipitation and noticed a reduction of the colon carcinoma in mice. PEGylation of the PHA nanoparticles proved to be critical in increasing the bioavailability of these DDS in selected applications. Lu and co-workers [92] found that a PHA copolymer (PHB-co-HH) when PEGylated as PEG-PHB-co-HH increased the delivery of a kinase inhibitor rapamycin. Since the regulation of kinase expression is one of the key targets in the prevention of the cell proliferation of malignant tumours, the aforementioned approach is particularly interesting. A modification of pegylated PHA-based DDS was reported wherein folic acid was conjugated to the system [93]. Folate receptor has shown its ubiquitous presence on the surface of cancer cells and it has therefore been regarded as a therapeutic target in the fight against tumour progression. Doxorubicin loaded folate conjugated PEG-PHA nanoparticles showed a significant 3.5-fold in vivo regression of the tumour volume in BALB/c nude mice compared to doxorubicin addition in free-form. A different approach that utilised PHA-based DDS encapsulated a hydrophobic photosensitizer (pTHPP) for a photodynamic therapeutic potential against selected cancer cells line (HT-29 cells) after 24 h [94]. There appears to be a direct correlation between PHA molecular weight and the controlled release of the photosensitiser and maximum photocytotoxic effect with respect to NPs of different polymeric formulations.

#### 3.1.3. Polylactic (PLA) and Poly-lactic-co-glycolic acid (PLGA)-based Nanomedicine for Potential DDS

BIND-014 is an engineered nanoparticle composed by a hydrophobic polylactic core and by a hydrophilic polyethylene glycol (PEG) outer shell decorated by a prostate specific membrane antigen (PSMA). In vivo studies of phase I clinical trials with patients having metastatic solid tumours adopted these BIND-014 nanoparticles loaded with a docetaxel drug and saw a retention of the NPs in the plasma compartment with limited clearance of mononuclear phagocytic defences. The PSMA decoration also allowed a specific targeting of the solid tumour cells [95]. From the results published by Von Hoff and co-workers, it is clear that using a nanoparticle-based delivery system significantly improved the therapeutic potential of the drug compared to the use of the free form of docetaxel. PLGA-PEG nanoparticles, containing two drugs, cisplatin and paclitaxel, as an aid for chemoradiotherapy against non-small cell lung cancers, showed greater tumour inhibition compared to the single-loaded nanoparticle or the drug administration in a free form [96]. Human oral squamous carcinoma cell lines, such as PE/CA-PJ15, were targeted in vitro by a study performed by Cacciotti et al. 2018 [97]. In this study PLGA nanoparticles and PLA nanofibers efficiently trapped and released in a controlled manner 18-β-glycyrrethic acid, a ROS promoter compound from liquorice plant. This compound specifically reduced the viability of PE/CA-PJ15 cells compared to non-cancerous human gingival fibroblasts. Intracranial glioma treatment can show poor outcome due to the blood brain barrier effect that decreases the therapeutic potential of the drug. One of the main challenges are premature drug degradation and low retention time in the cancerous tissue. A proposed solution could be the use of hydrophobic nanoparticles based on PLGA for the safe delivery of the drug. Householder and co-workers [98] used camptothecin (CPT)-loaded PLGA nanoparticles for an intravenous administration to target intracranial glioblastoma multiforme (GBM). The hydrophobic nature of the drug in combination with the hydrophobic nature of the nanoparticles improved the survival rate of the mice bearing this type of tumour. In fact, the 1.3-fold life expectancy extension was seen after administration of CPT nanoparticles, as well as a 2-fold decrease of the tumour size. In addition, the hydrophobicity of the nanoparticles allowed higher drug loading that could also promote a better controlled release at concentrations comparable to those in the free-from. Similarly, increased hydrophobicity of the drug specifically targeted the core of the GBM, preventing enhanced permeability and retention (EPR) by extravasation from leaking tumoral vasculature. Intravenously supplied cytarabine loaded PLGA was taken up by the key organs that leukemia cells affect because of blood circulation, therefore showing efficiency compared to the drug supplied in a free-form [99]. PLGA/PEG folic acid conjugated (PPF) nanoparticles with nanoencapsulation of curcumin as chemosensitizer/therapeutic agent in combination with or without paclitaxel showed a marked reduction of cervical cancer xenografts in mice [100]. Genexol-PM is a polymeric micellar compound, in which mPEG-PDLLA acts as a solubiliser of the paclitaxel drug for chemotherapeutic treatment of cancer. Ahn and co-workers [101] performed a phase II clinical study where gemcitabine was administered in patients with advanced non-small cell lung (NSCLC) cancers. The response rate was 19% higher than Cremorphor^®^-paclitaxel drug-form, and myelotoxicity and emetogenicity were also lower. Tamoxifen loaded PLA nanoparticles showed a minimally better outcome in breast cancer reduction in mice in respect to the use of tamoxifen on its own, but both hepatotoxicity and kidney toxicity were reduced with the use of PLA NPs in addition to reduced oxidative stress [102]. mPEG-PLLA nanoparticles were also used for the treatment of breast cancer, but they were loaded with docetaxel, and a more hydrophilic segment (poly-L-lysine) was introduced to create MPEG-PLLA-PLL micelles MCF-7 [103]. These were then administered to mice model and caused shrinking of the subcutaneous breast cancer. The hydrophilic portion of the micelle and the hydrophobicity to hydrophilicity ratio allowed a marked increase in the drug efficiency loading and a target delivery in the cancerous tissue. Giteau and colleagues [104] achieved an 80% encapsulation efficiency using protein precipitation and its subsequent encapsulation solid by emulsification of PLGA microparticles in oil in water (S/O/W) nanoemulsion. After the lysozyme treatment, no loss of activity after precipitation and encapsulation was detected.

Even though PLA and PLGA nanoparticles showed good biocompatibility and bioavailability in a variety of drug delivery systems, two issues arise with their use. One is the stability of the loaded drug due to ionic interaction between the carboxy terminus of the biopolymer and the N-terminus of a protein-based drug [105,106]. The second is the formation of acidic residues (glycolic and lactic) in an aqueous environment, due to the erosion of the nanoparticle. These can lead to either delayed or uncontrolled release [107,108].

#### 3.1.4. Uncommon Biobased Biopolymers for Potential DDS

Poly-γ-glutamate acid is usually produced by fermentation of Gram-positive bacteria. It is a highly hydrophilic polymer that given its superior water-solubility has been studied for DDS. For example, Khalil and colleagues have studied it as a system for the encapsulation of adenovirus as an oncolytic adenoviral vector to protect the load against neutralizing antibodies of the immunogenic response [109]. Ashiuchi and co-workers studied the opposite effect, where PGA coating was intended as antiviral effector to protect different surfaces [110]. They demonstrated the viability of this system against influenza virus as well as against fungal and bacterial pathogens. Lorens et al., 2015 [111] focused on the use of PBS-based DDS and saw that anticancer drugs curcumin and triclosan could be successfully loaded and more importantly slowly released by PBS as the hydrophobic component of the DDS. 

#### 3.1.5. Stimuli-Responsive Biopolymer-Based DDS

When biopolymeric DDS are utilised for therapeutic purposes, one of the key features of these systems is to avoid a burst release of the loaded drug. One of the methodologies to prevent that involves promoting a stimuli-responsive effect, only within the target tissue. A typical approach to promoting a stimulus response degradation (SRD) is to introduce labile bonds in the polymeric assembly that can be cleaved in the target tissue; i.e., the cancerous cells [112,113]. A widespread approach to achieve it is to introduce a disulphide bond that can be cleaved by the oxidation of glutathione [114]. Another approach is to introduce acidic-sensitive moiety in the micellar assembly to promote the SRD. Finally, an enzymatic driven degradation, for example, by an esterase, can be induced within the target tissue [115]. PLA is typically conjugated with an amphiphilic block copolymers (ABPs) to introduce hydrophilic residues in the hydrophobic structure of the biopolyesters. Having these PLA-ABPs (for example, PLA and PEG or PLA and PMMA) linked by a bromide disulphide-carrying oligo double-head hydroxide macroinitiator will afford a PLA-SS-POEOMA polymer that will be a hydrophobic-disulphide linked-hydrophilic. When this polymer self-assembles, it creates micelles that can be degraded by reduced glutathione in target cells and release the drug. This has been studied as a proof of concept and tested at the laboratory-scale [116]. Subsequently, Ko and co-workers [116] created an assembly where the hydrophobic PLA core carried doxorubicin drug, while the hydrophilic cationic outer layer was linked by electrostatic interaction to target specific oligonucleotides used for gene-silencing. The interlinking disulphide bonds were critical in allowing the double attack strategy. This was tested in vitro against HeLa cells. Variation of this PLA-SS-ABP strategy introduced H-bond facilitator molecules, such as amide units [117] or the use of triblock copolymers such as PEG-SS-PLA-SS-Folate with a redox-responsive assembly, used against cancer cells [118]; or the use of acid-labile, i.e., hydrolysable tetrahydropyran (THP) in a PLA-THP-PEG assembly [119]. Yu and colleagues [120] directly conjugated the doxorubicin drug to the PLA polymer by a pH-labile linkage, a strategy proven to be successful against breast cancer cell lines. A physical stimulus such as temperature change or electromagnetic induction is also a potentially swift solution as an efficient nanomedical therapy. Thermo-responsive PLA has been used by Jain and co-workers [121] in a nanoparticle loaded gel-form for mucosal absorption and then glioma delivery of methotrexate anti-cancer drug. Even if it is not clear how the thermo-response of the gel acted, it looks like an increased stability of the gel against mucosal clearance allowed controlled release of the drug in mice models. A similar use of a thermo-responsive PHA-based gel (i.e., PHB-PNIPAAM-PEGMEMA) assembled in a multi-arm fashion, capable of increasing the swelling potential and the thermo-response of the gel, for a controlled drug release at human body temperature (37 °C) was developed by Barouti and colleagues [122]. The use of doxorubicin loaded in the gel was tested with human fibroblast cell lines. A quadriblock system of PHBH/PEG/PPG/urethane where PHBH represented the hydrophobic moiety, while PEG and PPG represented the hydrophilic and thermolabile segments respectively, allowed an efficient docetaxel drug loading and melanoma reduction in xenograft of mouse models [123]. A more elegant and ambitious approach is the use of photo-responsive polymers as DDS. Two inherent challenges are associated with this choice: (1) photo-responsive molecules that are conjugated with a micellar polymer are not necessarily biocompatible, and can in some cases induce non-specific cytotoxicity and (2) electromagnetic penetration is limited in most of the tissues strictly correlated with the wavelength of choice [124]. A common approach involves photo-isomerisable molecules such as spiropyrane or azobenzene moieties. These photo-isomerisable molecules can induce a drug release from loaded micelles by promoting micellar disruption or micellar deformation [124]. For example, stearic acid linked to a PEG segment via a UV-responsive nitrobenzaldehyde (NB) linker elicited a cytotoxic effect in breast cancer cell lines [125]. A clever approach, performed by Yan and colleagues [126], to overcome the limited penetration of UV excitation was to dope polymeric nanoparticles with lanthanides-based molecules that respond to a near infrared (NIR) excitation and cause a localized UV emission. However, this just demonstrated proof of concept and did not have any in vitro or clinical in vivo testing. This pioneering study was followed by more complete studies that exploited the same principle but improved the design of the nanoparticles or the payload of the nanoparticle and went ahead to test an in vivo effect of this DDS to advance the technology. Wang and co-workers [127] used polymethacrylic acid lanthanides loaded nanocapsules for the delivery of doxorubicin by a NIR trigger. This is a better design of nanoparticles; however, no in vitro or clinical in vivo testing was conducted. An even more complex core-shell nanoparticle where a lanthanide-based nanocapsule, with a photosensitiser drug hypocrellin A loaded above a silica nanoparticle and wrapped by an outer shell of modified PEG-folate for the concomitant loading of si-RNA was used for gene silencing in HeLa cells or tumour bearing BALB/c nude mice in vivo. In both cases a therapeutic improvement was seen using this engineered system [128].

**Figure 3:**
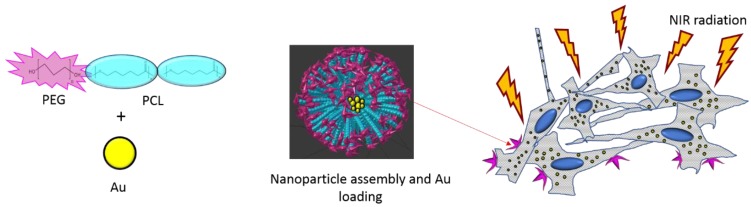
Polyethylene glycol-polycaprolactone (PEGylated-PCL) micelle loaded with gold nanoparticles to induce apoptosis into NIH3T3 cancer cells in vitro, when stimulated by near infrared radiation (NIR). *Freely adapted from Shagan et al. 2018* [129].

As evidenced from the above examples, to achieve this photo-responsive DDS, neither the photoresponsive molecule nor the nanocarrier were biobased or fully biocompatible, which limited application in vivo. Few examples are available where simplicity of design joint with truly biocompatible and biodegradable materials led to the design of photo-responsive DDS. PEG/PCL biocompatible and biodegradable polymer loaded with gold nanoparticle and triggered by a NIR light driven thermogeneration was shown to be active against NIH3T3 cells in vitro (Figure 3) [129]. Bupicavaine as a drug model was also successfully tested with this system. A paradigm shift of the use of PLA-based materials for waveguide-based technology instead of a DDS photo-responsive nanoparticle was achieved by Nizamoglu and colleagues [130], where photochemical tissue bonding, a carefully designed device to deliver phototerapeutic laser treatment in the subdermal section of a living organism, was used to achieve a 10-fold increase in visible light penetration using a biodegradable and expendable waveguide, which promoted a more efficient closure of the wound when suturing with this technique.

An advantage of polymeric-based DDS is that different chemical modifications can be achieved given the wide-range of functionalities that can be introduced into the polymeric structure; therefore, in principle, a more tailored approach can be envisaged for controlled drug delivery in tissue-specific approaches [131].

#### 3.1.6. Biopolymers for Biophotonic Applications

A cross-disciplinary section between the use of biodegradable polymer for DDS and for tissue engineering is the use of these polymers for biophotonic applications. In fact, the photonic-driven technology, in the therapeutic space, can be considered as a bridge between a DDS (specifically photo-responsive) and implantable devices for tissue engineering [132]. Shan and co-workers [133] used a citrate-based polymer as a biodegradable and implantable optical fibre with minimal loss in refractive index. As a proof of concept, both fluorescence emission via light induction and image transmission via optical fibres were successfully achieved in a rat model. The researchers recommended careful consideration of the correlation between the bending of the implanted optical fibre and the resolution power of the retrieved image. An inversed biomimetic approach, where a technology is mimicked by biological components, was proposed by Nizamoglu and colleagues [134]. These researchers devised a biolasing technology using a flavinomononucleotide (FMN) as an excitable molecule and achieved the lasing effect by a careful mirroring of the induced fluorescence by two portions of super hydrophobic PLLA resonators. Kim and co-workers [135] used a PLLA-based optical needle against skin cancer in a photodynamic therapeutic setting, achieving a 9-fold improvement in light delivery using this biodegradable device; additionally, blue-light delivery for antimicrobial effect was achieved. Pramual and colleagues [94] were the first to attempt the use of PHA nanoparticles as drug-carriers for photodynamic therapy (PDT). As both the PHA nanoparticle and the photosensitizer (pTHPP) are hydrophobic, it was quite easy to achieve a stable construct by mixing these two. The human colon adenocarcinoma cell line HT-29 was the tested target to see the controlled release of the photosensitiser drug. The drug release was 100% after 24 h and the photocytotoxicity was 90% at 6 h. The slow degradation rate of PHA nanoparticles outperformed similar constructs made of PLGA nanoparticles, where the degradation was faster, but the risk of burst release was significantly increased. In general terms, even if the promise of this technology is highly appealing both for elegance and precision, the use of bioplastics-based photonic technology is still mostly a matter of research and far from a commercial reality.

### 3.2. Biodegradable Polymers as Devices for Tissue Engineering

In tissue engineering applications, biopolymers have proven useful in replacing biogenic materials that could induce an immunogenic reaction due to non-specific host response [136,137,138]. Polymer composition and the possibility of introducing reactive functionality widens the potential of using biopolymers in this field [139]. While features of biopolymers such as hydrophilicity, biodegradability, biocompatibility, porosity, and non-toxicity make them attractive materials in many biomedical applications; hydrophobicity combined with biocompatibility and non-toxicity could be determinant in some selected applications where hydrophobicity is a key advantage [140,141,142]. On the other hand, the processing of the polymer and the device’s design are equally critical for successful tissue engineering applications [143,144,145,146,147]. 

The most widely used biopolymers in tissue engineering are PDLLA, PLGA, PHA, PBS, Poly-γ-glutamate. Both PLLA and PLGA have interesting applications in tissue engineering due to their range of melting temperature and glass transition temperatures; for this reason, they have been extensively considered for tissue engineering applications, with some of them being commercially available [148,149,150]. The downside of these polymers is that during the breakage of the ester bond by hydrolysis, many acidic residues are generated, and this could have an impact on the physiology of the cells or the target tissue. 

#### 3.2.1. Bioscaffolds for Cell Cultivation In Vitro

PLLA proved particularly effective in inducing positive outcomes in osteogenesis in vitro. Gutierrez-Sanchez and colleagues [151] functionalised PLA scaffolds through adsorption of a tripeptide moiety that facilitates cell adhesion. Similar adhesion results were seen by osteoblasts with or without the tripeptide functionalisation. Lee and co-workers [152] performed a more complete study wherein they took into account the detrimental effect that PLGA could have due to the release of acid residues during biodegradation. They therefore developed a composite wherein calcium phosphate and magnesium hydroxide were added to a PLGA scaffold to neutralise the acid residues upon their release. This led to a distinctive osteoclastic differentiation as well as to a reduced immunogenic effect by magnesium hydroxide, and a better mechanical and physiological response induced by the PLGA/calcium phosphate composite. The processing of the starting polymer was shown to be critical in inducing specific outcomes for the plated cells, as shown by Kareem and colleagues [153]. They have found that a core-shell electrospinning to fabricate a PCL/PLLA scaffold with hydroxyapatite particles allowed an osteoconductive environment and also induced differentiation of human mesenchymal stem cells (hMSCs). A similar study by Xu and co-workers [154] used a PCL/PLA electrospun composite, but they specifically saw that an increase in the PLA content induced a higher alkaline phosphatase and calcium deposition due to the higher stiffness of the scaffold. The same group also saw a higher osteogenic differentiation of hMSCs in vitro and more functional cranial bone formation in vivo due to this PCL/PLA blend [155]. Mineral composites are particularly critical for the osteogenesis process in vivo. Specifically, calcium deficient hydroxyapatite (dHA) induces a superior calcium and phosphate decrease, triggering a cascading osteoinductive effect that involves a fine-tuned immunoresponse [156]. Mineral/biomaterials composites having the benefit of the osteoinductive potential (from the mineral component) have superior biocompatibility (induced by the biopolymer); this double feature favours the cross-talk between the substrate and the mammalian cell. Bianco et al. 2011 [157] and D’Angelo et al. 2012 [158] showed that electrospun PLLA/dHA could differentiate toward the osteogenic lineage human (bone marrow derived) hMSCs or even induced pluripotent adult or embryonic stem cells (iPSC and ESC) of murine model. hMSCs were also used by Zhang and colleagues [159] to induce the production of neurotrophic factors after the successful plating on DOPA-IGF-1 bound PLGA scaffold. The successful production of neurotrophic growth factor allowed PC12 cells to have a sustained neurite outgrowth. Liu and co-workers [160] focused on the in vitro growth of hepatocytes and saw that a lecithin modified PLA-polyurethane composite allowed HePG2 cells to grow more sustainably compared to growth in culture plates alone. A more peculiar approach was reported by Paolini and colleagues [161], wherein a PLA polymer was coated with a polyamidoamine bound miRNA to transfect genes to plated HeLa cells. Even though this was more of a proof of concept, the system showed potential to use these polymers as a template to control cellular metabolism by transfection of genes. 

Petriz-Reyes and colleagues [162] modified PHB scaffolds with polyurethane (PU) residues. When mouse astrocytes, from cerebellum and human embryonic kidney cells (HEK293), were plated on these 3D PHB-PU scaffolds, they showed a successful intracellular Ca^2+^ signalling, indicating survival of the cells. Kwiecien and co-workers [163] condensed sebacoyl chloride with oligomers of PHBH, achieving PHB-co-HH-co-SEB. This particular terpolyester allowed successful plating of HEK293 cells and fibroblasts. A fully PHA-based scaffold where PHB represented the stiff component while *mcl*PHA represented the soft segment, named binary PHA, was designed by Lukasiewicz and colleagues [164]. This composite was extremely effective in promoting the proliferation of myoblasts cells, potentially due to the increased softness that the *mcl*PHA provided to the composite. PHB has also proven successful at promoting neural cell growth. It has been effective using neural stem cells [165,166], cortical neurons [167], or differentiating human bone marrow stem cells into nerve cells [168]. A paradigm shift wherein *mcl*PHA took the lead among the PHA material for soft tissue engineering, has been promoted by Ipsita Roy and collaborators since 2011 [169]. A few examples are sufficient to identify the specific potential of *mc*l-PHA in this field. The cardiac regenerative potential of *mcl*PHA to promote the proliferation of neonatal ventricular rat myocytes was demonstrated by Bagdadi and colleagues [170]. Constantinides and colleagues [171] used a PHA/PCL blend for cardiac stem cell cultivation and implantation in animal models of infarcted mice, thereafter seeing a critical improvement of these scaffold for cardiac cell regeneration in vitro and in vivo. It looks like both biocompatibility and mechanical properties are determinant in promoting the cardiac regeneration mediated by these scaffolds. Rai and colleagues [172] also attempted the plating of keratinocytes for skin-regeneration technology on these *mcl*PHA scaffolds. *Mcl*PHA polymeric films blended with bioactive glass nanoparticles enabled keratinocyte proliferation in vitro. 

PBS polymers, despite their softness and higher elongation at break, could be hindered by their smoothness, in tissue engineering applications. Patntirapong and co-workers [173] promoted a sodium hydroxide hydrolysis of PBS/calcium phosphate composites and saw a concentration-dependent increase of vinculin and actin-reorganization-mediated adhesion of hMSC to be differentiated in osteogenic cells. Liverani and colleagues [174] also used PBS-based polymer electrospun with softer segments of dilinoleic succinate and/or polyglycerol sebacate, creating fully biobased biopolymers. These were then tested on mouse skeletal myoblast cell line C2C12 and on mouse postnatal cardiomyocytes. However, the results for cell adhesion or polymer characterisation were not extensively detailed, but according to the authors they looked promising. Jager and colleagues [175] explored further PBS polymers as well as poly(alkene) succinate derivatives and found that PBS shows a high biocompatibility with hMSC favouring their adhesion and proliferation. They also found that polyethylene succinate (PES) has a successful outcome when used as an antifungal polymer. Similarly, Fabbri and co-workers [176] improved PBS-based polymer with PEG segments to increase the hydrophilicity of the materials and quite considerably increase the adhesion of embryonic rat cardiac H9c2 cells. This was proved by a higher expression of the heavy chain of the myosin protein as a commitment towards a muscular feature of the cells. The benefit of integrity given by hydrophobic polymeric scaffolds is lost by biobased polymers of microbial origin such as polyglutamic acid (PGA), that are more hydrophilic, and therefore more attractive for cell adhesion but more prone to hydrolysis if not modified. Gentilini and colleagues [177] esterified the exposed carboxylic of PGA with benzyl residues and saw a 3-fold higher viability of hMSC cells compared to PLLA scaffolds. Therefore, they concluded that benzyl-modified PGA gained in hydrophobicity, but still allowed excellent cell adhesion on PGA scaffold. Clarke and co-workers [178] enhanced the hydrogel-like characteristics of PGA by cross-linking the β-sheets to tailor the stiffness, reducing in a tailored way, the hydrophilicity of the hydrogel and also allowing a self-healing mechanism through the recovery of the original mechanical properties. 

#### 3.2.2. Bioplastics Polymers as Implants In Vivo

A quite successful polymer for in vitro applications could turn out to be ineffective once used in vivo. Very few examples of commercially available biopolymers are used for in vivo applications, with most of them being based on hyaluronic acid or collagen polymers [179]. A way to get around the reduced biocompatibility of biopolyesters, for example, PLGA, is to create composites that incorporate extracellular matrix (ECM) elements. Lih and colleagues [180] increased the concentration of ECM in PLGA scaffolds and successfully cultivated renal cortical epithelial cells and implanted these devices into nephrectomised mice, assisting in the recovery of blood vessels as well as reconstitution of glomeruli in vivo. This result could represent an engineered way to remove the need for dialysis for kidney impaired patients. Biobased polymers have often been considered preferable materials for two kind of implantable medical devices: adhesion barriers and stents. The former are used in post-surgical scenarios to reduce tissue adherence and inflammation. The latter are instead used to maintain the blood flow in diseased arteries, typically after thrombotic events or to prevent a build-up of arteriosclerotic plaques. Yamaoka and colleagues [181] substituted the epithelial tissue around the caecum and the pericardium around the hearth of Wistar rats with electrospun membranes composed by different PLA/PEG ratios. PLA/PEG (68/32% w/w) and PLA/PEG (12/88% w/w) were found effective in avoiding the post-surgical adherence of the caecum and the hearth. Ma and co-workers [182] tested a PLA composite with hydroxyapatite in vivo on a rat mandible defect model. The PLA scaffold avoided soft tissue adherence on the mandible, but it was not so effective for human adipose derived stem cells’ (hADSCs) osteogenic differentiation in vitro. Somekawa and colleagues [183] used PLA to make an aqueous suspension have a sol–gel transition for gel behaviour at body temperature. This gel composite proved highly useful in preventing left ventricular remodelling after myocardial infarction in rat models. Since stent devices are inserted into arteries to maintain blood flow, two desirable features should be promoted by them: an effective widening of the collapsed arteries and the prevention of restenosis, i.e., the recurrence of an abnormal narrowing of the intervened arteries). Biopolymeric stents are currently on the market and they are competitively showing their potential. PLA-based stents are the major actors of the stents market. Some of the commercially available PLA-based stents are the following: Igaki-Tamai^®^ by Kyoto Medical Planning, DeSolve^®^ by Elixir Medical Corporation, Mirage BRMS® by Manli Cardiology, Absorb GT1^®^ by Abbott, MeRes^®^ by Meril, Xinsorb^®^, ART 18AZ^®^ by Arterial Remodelling Technologies and Nobori^®^ by Terumo. All of these are bioresorbable in a range of time between 18 and 24 months and all of them are drug (Sirolimus^®^ or Myolimus^®^)-eluting stents wherein the drug serves to prevent restenosis. 

Despite the continuous improvement in polymer processing and medical device design, most of these biopolymers are underperforming in respects to the metallic stents, especially in terms of elongation at break and stiffness [184]. However, they outperform metal stents in biocompatibility, biodegradation, and reduction of arteries ruptures. For this reason, a continuous improvement in bioresorbable stent research is ongoing. This improvement is trying to address these shortcomings, especially in polymer processability and composition, medical device design, and drug delivery combination. Kum and colleagues [185] proposed using magnesium oxide along with PLA in composites that in a biodegradation scenario could buffer against the acidic residues arising from the released lactic acid. These composites also showed interesting mechanical properties and therefore were proposed as suitable for implantable biopolymers. The performance of PLA drug eluting stents was found as positive in a one-year clinical trial [186], but they also showed limitations when implanted in a suboptimal way [187]. Given problems in recoil of the polymeric stents and device induced thrombosis, the Nobori^®^ developers opted for a solution that combined chromium-platinum stents with a PLA coating that could elute anti-restenosis drugs [188]. Nikoubashman and colleagues [189] tested PLA composites for stent application, specifically for neurovascular surgery with a PLA-co-GA copolymer, where 85:15 proved to be effective for such an application. However, this composite induced diffuse foreign body reactions and granulocytic inflammations in porcine animal models, thereby posing a barrier for in vivo implantation [190]. Arai and colleagues [191] encapsulated basic fibroblast growth factor (bFGF) and agratroban in the PLGA coating of a metallic stent and tested it in a rabbit model to positively overcome the effect of an induced aneurysm. To the best of our knowledge, the only in vivo study of a prosthetic cardiac valve made of polyglycolic acid and polylactic acid in a 50:50 ratio was reported by Gottlieb and colleagues in a sheep model [192]. The implantation went according to the intended plan, but a pulmonary regurgitation was seen after 20 weeks of follow-up.

**Figure 4:**
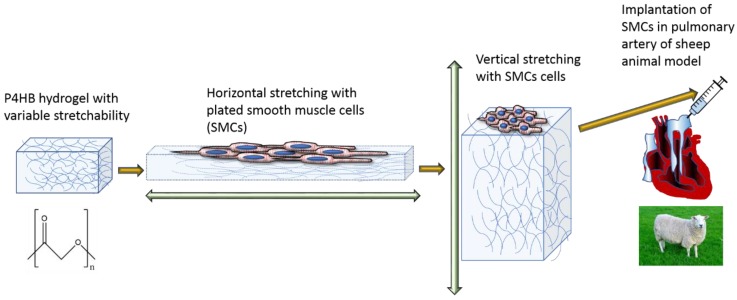
Poly-4-hydroxybutyrate-based hydrogel as an anelastomeric scaffold for a cyclic stretchability for the improved plating of smooth muscle cells (SMCs). These cells were implanted on a pulmonary artery of an animal model (sheep) after the growth on the scaffold in vitro. *Freely adapted from Masoumi et al. 2017* [194].

Poly-4-hydroxybityrate (P4HB) is the only suitable PHA to show the required mechanical properties for a wide range of applications in the medical sector. Tepha Inc is one of the most active and early adopters of polyhydroxyalkanoates-based technology for the medical sector [193]. Masoumi and colleagues embedded a P4HB polymer into a photo-crosslinkable hydrogel with an elastomeric gradient of behaviour (anelastomeric, Figure 4) [194]. This composite was cultivated in a cyclic stretch/flexure bioreactor to train the device, with seeded smooth muscle cells (SMCs) and then implanted in the pulmonary artery of a sheep model. The device performed greatly with no thrombogenic effect and progressive in-growth of host tissue.

Collagen-coated melt electrospun and braided P3HB with seeded hMSC were implanted as the vector to deliver the precursor for osteodifferentiation. The implantation into immunodeficient nude rats induced vascularisation and effective osteodifferentiation but was not sufficient for new bone formation [195]. Webb and colleagues reconstructed a severed tendon with a PHB-co-HH coated with collagen in a rat model and saw no inflammation after 40 days of implantation [196]. Gredes and co-workers successfully implanted PHB film patches into mice bearing cranial-defects [197]. These patches promoted bone formation in 50% of the cases and considerably increased blood vessel development and outperformed the bone formation of untreated mice. George Guo-Qiang Chen’s group observed that 3-hydroxybutyrate (3HB) monomers leaking after hydrolysis of implanted PHB promoted calcium deposition and alkaline phosphatase in murine osteoblasts [198]. Wei and colleagues used PHA as porous microparticles as an injectable scaffold for hMSC delivery [199]. These hMSC differentiated in vivo in a mouse model and gave rise to ectopic bone formation. This solution outperformed hydrogel as a cell carrier, yielding maybe the most effective PHA-based technology for tissue regeneration in vivo to date. 

A niche sector where PHA materials are preferred as a material of choice for in vivo application by researchers is neurite guidance for peripheral nerve regeneration. Many examples of PHA materials as heterologous nerve xenografts can be reported; among them is the study of Sakar and colleagues [200], where PHB was loaded with hMSC for peripheral sciatic nerve regeneration in a mouse model. 

The most pioneering work for the use of PHAs in vivo for cardiac engineering applications was performed by Sodian and co-workers [201], where an autologous population of endothelial cells from a carotid arteria and a jugular vein were seeded in a tri-leaflet heart valve model made of porous polyhydroxyoctanoate and re-implanted into a lamb model. Up to 17-weeks of follow-up was performed, with a healthy spectrum of data for the animal after the surgical procedure of re-implantation. Wu and colleagues [202] followed up on this pioneering study by using a reversed approach where the decellularised original heart valve was coated with PHA, and they tracked the confluency of the cells on these hybrid valves after implantation. The mechanical performance of these hybrid valves was higher while the calcification was lower. While PGA has been used in vivo as nanocapsules for therapeutic gene or for anti-cancer drug delivery in mice model [203,204,205], it has not been tested as a scaffold in vivo. 

PCL outperforms all the above-mentioned polymers in terms of viscoelasticity and rheological properties [206], and it is especially sought after for in vivo applications. Woodruff and colleagues [206] provided a detailed review dedicated to PCL and highlighted its superior characteristics. Here we try to narrow the focus in examples of outstanding in vivo applications where PCL is the polymer of choice. Two PCL-based polymers Osteopore™ and Artelon^®^ are used for osteoregeneration. Huang and colleagues [207] recruited PCL as a fibroid material to promote chondrocyte regeneration and therefore prevent surgical operation in this difficult to regenerate tissue (typically, in cases of osteoarthritis). A similar in vivo study was reported by Shao and colleagues [208], wherein bone marrow derived mesenchymal stem cells (BMSC) were seeded on PCL scaffolds implanted on the site of a chondro-induced defect in a rabbit model. There was a positive outcome still after 6 months of follow-up study. Li and co-workers [209] saw a very similar result in a swine model. PCL was initially considered as prosthetic for the replacement of the aortic or mitralic valve to correct heart defects. A mixture of electrospun (to allow cell invasion) and knitted (to give strength) PCL seemed the optimal combination identified by Van Lieshout and colleagues [210]. The ability to have contractile movements for cyclic behaviour of contraction/extension for prolonged periods of time, is one of the key features of biomaterials for graft vascularization and blood vessels regeneration. Jeong and co-workers [211] identified a copolymer made of PCL and PLA (called poly-L-lactide-co-ε-lactone, PLCL). This polymer has specifically the right mechanical properties to sustain cyclic deformation and improved elongation at break to be considered for cardiac applications. This polymer has been tested in mice model, with the seeding of smooth muscle cells (SMCs) and these devices showed markers of healthy SMCs and improved biocompatibility for in vivo studies. This tissue-engineered vascular graft (TEVG) with a coating of polyglycolic acid was also tested by Brennan and colleagues in lamb models [212]. Immunohistochemical staining, qualitative biochemical analysis, superior vascular regeneration, sustained collagen, and elastin generation are all key signatures of a positive outcome. 

Lorden and colleagues [213] used PLCL for skin regeneration and reduction of hypertrophic scars. In vivo studies in a mouse model found a reduction in hypertrophic scar contraction when treated with PLCL. This is specifically attributed to the superior elastic behaviour of this polymer. 

**Figure 5:**
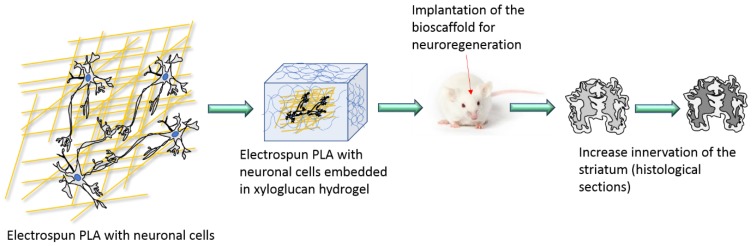
Electrospun polylactic acid (PLA) loaded with neuronal cells and embedded into a xyloglucan hydrogel for the neuro-regeneration of mice animal model by increasing the innervation of the striatum tissue of the brain. *Freely adapted from Wang et al. 2016* [219].

Neurolac^®^, a commercial polymer made of PDLLA-co-PCL (65:35% w/w) not only showed peripheral nerve regeneration but also axonal guidance in the central nervous system; Nisbet and colleagues [214] showed axonal penetration and scaffold-neural integration with no macroglia inflammatory response or glial scars in rat brains. A long-term study (2-years) was also performed with this material and showed no evidence of foreign body reaction after full resorption of the scaffold [215]. However, minimal microscopic fragments of the scaffold appear to be co-located with macrophages. Various modifications of PCL-based scaffolds have been tested *in vivo,* intended to reduce the inflammatory response and the detrimental microglial response for both nerve regeneration and central nervous system regeneration (by axonal extension and neurite elaboration). Suzuki and colleagues [216] tested methylcobalamin, vitamin B12 variant-doped PCL electrospun fibres, and saw no electrophysiological or histochemically detectable detrimental effect in sciatic nerve injured rats. Potas and colleagues [217] doped electrospun PCL fibres with interleukin-10 (IL-10) to increase its bioactive half-life and deliver a stimulatory response around injured sciatic nerves of Wistar rats. This induced a polarizing effect for macrophages in vivo after at least 14 days. Lee and colleagues [218] adopted an injectable PCL/hyaluronic acid hydrogel composite as the carrier of neural stem cells and successfully delivered it to an animal subjected to the middle cerebral artery occlusion (MCAO) stroke model, resulting in a full recovery—blocking microglial infiltration and reducing the inflammatory response. A similar injection approach was undertaken by Wang and colleagues (Figure 5) [219], wherein a PLA/hydrogel composite loaded with glial-derived neurotrophic factor (GDNF) implanted in the brain showed no deleterious impact on the host immune response, enhanced the survival of ventral midbrain grafts, and reinnervated the striatum. This study gives particular hope for an animal model of a Parkinson’s disease recovery.

Maclean and co-workers [220] found that galactose modified poly-lysine organised into a layer-by-layer (LbL) morphology with PCL, successfully promoted survival of neurons after traumatic injury in C57/BL6 mice model. This was also correlated with an increase of astrocytes around the scaffold after implantation. This is thought to be mediated by galectins that bind specifically to the galactose moiety and therefore reduce the inflammatory response of the tissue.

## 4. Bioplastics and Biodegradation

The need to protect our environment against plastic pollution, and to use renewable resources, i.e., biomass, waste, etc., to fulfil our material needs has gained worldwide attention in recent years. While biobased and biodegradable plastics can offer some technological innovations, they should not be looked at as a holistic technological solution to our environmental and sustainability concerns. As was already mentioned, all bioplastics are produced using natural resources, but the biobased origin does not mean that biobased plastic is also biodegradable. Biobased polyethylene (bio-PE), polyethylene terephthalate (bio-PET) and polyethylene-2,5-furandicarboxylate (PEF) are chemically the same or similar to crude oil derived PET and PE, and they are not biodegradable [221,222]. TPS, PLA, and PHA on the other hand, are biobased and biodegradable. However, whether a material will completely biodegrade (i.e., be mineralised) in a certain environment depends on its characteristics, such as crystallinity, but also on the presence of additives, such as plasticisers, and the environmental conditions where the plastic is located; i.e., presence of adequate microorganisms, temperature, moisture, and pH [223]. Thus, a bioplastic might be biodegradable in some but not all environments. PLA for example is highly crystalline which gives it desirable properties for applications. However, higher temperatures are required to allow biodegradation. PLA reaches international standards for biodegradation in industrial composting and anaerobic digestion conditions, but shows a very slow rate of degradation in soil and water, with estimates being that it would take decades or longer to completely degrade depending on the environment [1]. TPS and PHB were the only bioplastics that satisfied the criteria for biodegradation proposed by international standards across various environments representing managed (industrial composting, home composting, and anaerobic digestion) and unmanaged environments (soil, fresh, and sea water) [1]. Blending of polymers with the aim of improving mechanical properties could have a positive/synergistic effect on their biodegradation capacity, but it could also decrease the biodegradation potentials of individual components. For example, blending of PLA with oil-based, but biodegradable polycaprolactone (PCL) resulted in improved mechanical properties and biodegradability of PLA under home composting conditions [1]. Similar to this, blends of PHB and PCL, and PCL and TPS showed higher degradation in anaerobic digestion, while the same blends exhibited antagonism during biodegradation tests in aquatic environments.

## 5. Biodegradable Polymers in Packaging

Materials like glass, paper, wood, metal, and plastics are used for primary, secondary, or tertiary packaging [224]. In primary packaging the material is usually in direct contact with goods, while secondary and tertiary packaging are used to assist the transport of the primary packed goods. As already stated, approximately 40% of the produced plastic goes to the packaging sector and it is mainly used as primary packaging [225]. Replacing conventional oil-based packaging materials such as PP, PE, and PET with bioplastics is seen as a solution to environmental problems and our dependency on crude oil. Use of biobased polymers for food packaging applications is steadily growing: out of about 2.11 million tonnes of bioplastic globally produced in 2018, 65% of the volume was destined for the packaging market [226]. However, when compared to thermoplastic synthetic polymers, biobased polymers suffer from issues such as high price, challenges in processing using traditional technologies, and inferior performances in terms of functional and structural properties [227]. As a consequence, even though there are examples of the use of bioplastic in packaging, biopolymers have not yet found broad applicability in this area. 

There are biobased equivalents of popular fossil-based plastics, namely, bio-PE, bio-PP, and bio-PET, which have been used in packaging. While biobased, these materials are chemically identical to their oil-based counterparts, can be processed using traditional equipment, can be recycled in current recycling schemes (although the rate of recycling of these plastics is still very low) but they non-biodegradable. 

The reduction of greenhouse gas emissions in plastics production can be achieved by using biobased resources [228]. Furthermore, switching to biodegradable packaging opens up more end of life options. However, biobased plastics should not be an excuse for society to continue to engage in unsustainable activities such as over consumption of plastic and throw-away culture. These habits promote pollution and create resource inefficiency and value loss from the material. This section will give an overview of research strides towards fulfilling the potential of biodegradable polymers in packaging and showcase some successful examples.

### 5.1. Starch

Starch and its blends represent the highest share in the global production capacities of biodegradable plastics [43]. The sources of commercially important starch are corn, wheat, rice, potatoes, tapioca, and peas. Starch plastics were among the first commercialised biobased and biodegradable plastics [229]. Commercial starch plastics are developed mainly for film, injection moulding, and foam applications. Starch has been widely used in edible coatings; i.e., thin layers of edible material applied to food products with the purposes of extending the shelf lives and providing an effective barrier against hazards. Coatings reduce moisture migration, reduce gas exchange, delay the changes in structure, and maintain integrity of food.

The abundance, low cost, and biodegradability of starch make this material highly interesting for packaging. However, the innate hydrophilicity and brittleness are two main disadvantages that limit the application of starch. Native starch granules are disrupted using water, heat, and plasticisers such as glycerol [63], maleic anhydride [230], and citric acid [231]. The plasticisers displace strong interactions between hydroxyl groups present in the starch molecule with new hydrogen bonds formed between the plasticiser and starch. This leads to the formation of homogenous TPS with increased chain mobility, decreased glass transition temperature, and consequently improved ductile and extension properties.

Physical and chemical modifications are the main routes to improving the properties of TPS. Similarly to the strategies used to improve the PLA characteristics, blending of starch has been widely investigated to improve its water resistance and mechanical strength. Starch was blended with polyvinyl alcohol; polycaprolactone; PLA; and non-biodegradable petrochemical polymers polybutylene succinate-co-adipate (PBSA), polybutylene adipate-co-terephthalate (PBAT), and polyethylene (PE) [230,232,233,234,235,236]. The hydrophilicity of starch also represents a challenge when it comes to blending with hydrophobic polymers such as PLA. For example, to blend starch with PLA and maleic anhydride, Zhan and Sun first used an initiator, 2,5-bis(tert-butylperoxy)-2,5 dimethylhexane (L101) to improve the compatibility of PLA, starch and MA [237]. This improved adhesion between PLA and starch and resulted in a PLA/starch composite (55/45) with increased tensile strength and elongation. The strategies with which to improve the toughness of PLA/starch blends, i.e., additive plasticisation, mixture softening, elastomer toughening, and interphase compatibilization, were recently reviewed by Koh and colleagues [238].

Polyvinyl alcohol (PVA) is frequently used to prepare blends with starch. Hydroxyl groups present in starch and PVA form hydrogen bonds, which likely have a positive effect on the compatibility of the two polymers. When different ratios of starch and PVA were tested, it was found that with the increase of starch component crystallinity, tensile strength, elongation at break and Young’s modulus decreased, while water uptake at equilibrium increased [230]. However, at 50% starch content, the films had good flexibility, with the elongation at break more than 1000% and tensile strength of 9 MPa, superior to the commonly LDPE package films [230]. When the effect of the proportions of TPS and PVA on the functional properties of the resulting blends were analysed, it was found that the films containing 60% (w/w) PVA showed the best thermostability values and elongation at break, and lower water-vapour pressure [239].

Since starch is viewed primarily as a food packaging material, interesting breakthroughs were made in the area of active food packaging. Recently, Menzel, and colleagues reported the use of antioxidants extracted from sunflower hulls as additives in starch films to produce renewable food packaging materials [240]. The compression-moulded starch films showed antioxidant activity, and while substituting a portion of glycerol, used as a thermal degradation protectant, with the extract from sunflower hulls resulted in a decreased thermal stability of the films, the optical properties and glass transition temperature were not affected.

While PBAT is of petrochemical origin, it is biodegradable, and it was found that the blends of TPS and PBAT also have improved mechanical and rheological properties [241]. In addition, when cellulose nanowhiskers (CNW) were included in PBAT/TPS blends, morphological and water barrier properties of the films were improved, and the films exhibited antioxidant and antimicrobial activities [242].

### 5.2. Polylactic Acid

PLA is the second most produced biodegradable plastic heavily used in packaging sector [243]. Some of the largest PLA producers are NatureWorks, that produces a range of Ingeo PLA polymers from lactic acid, tailored for specific performance [244], and Total-Corbion [148] which produces a portfolio of PLA resins suitable for packaging and disposables, heat packaging and disposables, electronics, fibres and locomotives, and lactide monomers. The advantage of PLA is that it can be easily processed by injection moulding, film extrusion, blow moulding, thermoforming, fibre spinning, and film forming into many shapes and sizes [245]. PLA is already used in a number of packaging applications, such as fruit and vegetable packaging, disposable shopping bags, and cups [246]. 

PLA is rigid, brittle, is relatively sensitive to heat deformation, has low gas barrier properties, and is difficult to heat seal compared to competitor fossil plastics such as PET [247,248]. Some of these challenges can be addressed by blending PLA with other polymers, by using micro and nanocomposites, by coating it with high barrier materials, and by polymer modification [243]. The coating of PLA with a thin layer of poly(ε-caprolactone) (PCL), or poly(ethylene oxide) was seen to improve gas and water vapour barrier properties without affecting the visual appearance of the PLA films [249]. Since PLA is brittle, blending with other materials is used as a strategy to increase its toughness; i.e., the ability to absorb impact energy without breaking. The goal toughness of PLA will depend on its final application, and in packaging it would mean resistance to cracking when pressed or squeezed. Toughening of PLA could be achieved via plasticisation, copolymerisation, and melt-blending [250]. Ideally, the blending material should be compatible, biodegradable, and non-toxic, and it should significantly decrease the T_g_ of PLA. In an ideal case two miscible polymers would form a uniform, single-phase products, with physical properties between the two blending materials. However, in the majority of cases polymers are immiscible, and blending of such polymers forms an interphase which can but does not always negatively impact the characteristics of the resulting blend. For example, the addition of oil-based, but biodegradable polycaprolactone (PCL) to PLA at different loads improved the flexibility of PLA [1,251,252]. While PLA and PCL are immiscible their interaction lead to formation of specific morphology of uniformly distributed spherical structures within the PLA matrix, ultimately decreasing the stiffness of the resulting blend compared to neat PLA [1]. For the various PLA-based blends characteristics and potential applications, read a recent review by Nofar and colleagues [253].

The use of nanomaterials, i.e., structures with at least one dimension at 10^−9^ scale, is seen as beneficial as it usually leads to the dispersion of the nanomaterial into the polymer matrix, giving a high surface to volume ratio [254]. Cellulose nanocrystals and nanofibers have been extensively investigated as cheap, biodegradable, renewable, strong, and stiff replacements for silicates, carbon nanomaterials, and metals [255,256,257]. The main obstacle in the application of cellulose nanomaterials is their hydrophilic nature. The studies of the effect of the preparation method and filler content were summarised in a recent review by Mokhena and colleagues [255]. In general, when cellulose nanocrystals or cellulose fibres are used, the tensile modulus and tensile strength of cellulose/PLA composites increase with increase in the nanocellulose content; however, at the expense of elongation at break and hence toughness [255]. Nevertheless, with the possibilities of nanocellulose functionalisation, thermoplastic processing of cellulose/PLA composites seems to have good potential for large-scale production of materials with a range of applications, including packaging.

One of the functional properties of PLA that needs addressing to expand its application to include a number of hot food and beverage containers and packaging is heat tolerance. High mobility of PLA at T_g_ results in random ester bond breakage; i.e., random chain scission and self-catalytic degradation [258]. A range of nucleating agents, including phthalhydrazide [259], cyclodextrin [260], thermoplastic starch [261], cellulose [262], and others, were shown to increase the crystallisation rate, which in turn decreases the mobility of PLA and therefore makes it more heat resistant [263]. The chain motion of the polymer could be also restricted by fibre reinforcements [264], and the already-mentioned blending and compounding.

Another means to improve the physical and thermal characteristics of PLA is pre and post-polymerisation chemical modification. Chain extenders such as diisocyanate, epoxy resins, and others are frequently used to increase the molecular weight of PLA [265]. The post-polymerisation modification usually refers to cross-linking using irradiation, which reduces the mobility of PLA, thereby improving its toughness, processing performance, and thermal stability [266,267].

The application of supercritical carbon dioxide (scCO_2_) as a solvent to this process brings several advantages, including processing at moderately low temperatures, and the fact that scCO_2_ acts as a molecular lubricant and induces polymer swelling and foam formation [268]. By controlling the temperature or pressure during foaming with scCO_2_, it is also possible to tune the crystalline structure through the controlling of PLA and therefore its properties [269].

The concept of active and intelligent packaging refers to the incorporation of antioxidants and/or antimicrobials to packaging materials to extend the shelf-life, improve food safety, or improve sensory properties. Active packaging systems can either deliver a compound into the packaged food or the headspace, or remove undesired compounds from the product and its environment [270,271]. Antimicrobials and antioxidants can be directly incorporated into the polymer to create active packaging in which the bioactive substance interacts directly with the food or with the headspace, thereby reducing or completely inhibiting the growth of spoilage and pathogenic microorganisms [272]. PLA/PCL films impregnated with thymol or thyme extract are examples of active PLA-based packaging with antibacterial activity [273].

### 5.3. Polyhydroxyalkanoates

The range of properties, from brittle thermoplastics to glue-like elastomers, renders PHA applicable in a range of areas, including packaging. PHAs were seen as a potential replacement of PP, PE, and PS [274]. With over 150 monomers making PHAs, their crystallinity and elasticity can be tailored towards different products, from films to storage boxes. However mainly due to the production costs, PHAs represented only 1.4% of the globally produced bioplastic in 2018 [42]. Another attractive feature of PHAs is hydrophobicity, which means good water barrier property [275]. In addition, CO_2_ transport properties of specifically short chain length (*scl*) PHAs are similar to polyvinyl chloride (PVC) and PET [275]. Among PHAs, *scl*PHAs, with polyhydroxybutyrate (PHB) as a representative, are good candidates for packaging application due to their high plasticity and processability by extrusion, injection moulding, and thermoforming. However, similar to PLA, PHB, and other *scl*PHAs show in general low ductility and flexibility. Blending and addition of plasticisers was widely investigated to improve the mechanical properties of *scl*PHA towards packaging applications [276,277,278]. While some efforts resulted in a material with improved toughness, ductility, and water properties [278], it is hard to overcome the intrinsic aging effect based on secondary crystallisation, as well as slow crystallisation rate, resulting in easy fracture of the material [279]. There are some reports which suggest that certain plasticisers, such as tri(ethylene glycol) bis(2-ethylhexanoate), triethyl citrate, and tributyl citrate, have no or even negative effects on the mechanical performance of PHB [276]. One of the possible explanations is that the plasticisers were not incorporated adequately, suggesting the importance of selecting the appropriate plasticiser and processing technique in order to minimise the effects of ageing [276].

Another negative characteristic of PHB is its narrow thermal processing window [280]. Several mechanisms, including a random thermal degradation, auto accelerated transesterification and chain scission from crotonate chain ends seem to be responsible for this behaviour [280]. Inclusion of 3-hydroxyvalerate (PHBV) and 3-hydroxyhexanoate (PHBH) during biosynthesis [281,282], the addition of low molecular weight polypropylene glycol [283] or polyethylene glycol [284], blending with other polymers [278,285], and cross-linking [286] are some of the strategies used to improve the thermal processability of PHB. The addition of PEG1000 to PHBV-based matrices significantly lowered the stiffness of films which also became more extensible, thermo resistant, improved regarding water vapour permeability, and more resistance to aging [287]. The choice of a plasticiser will clearly depend on its effectiveness; on the rate of its volatility or migration during use; on the storage, toxicity, and renewability; and on how it affects the biodegradability of the final material. While bisphenyl A (BPA) was also shown to reduce the secondary crystallisation associated with ageing, BPA is toxic and therefore could not be used for packaging. Recently, Jenkins and colleagues have demonstrated that saccharides could be applied instead of BPA [288]. While the addition of saccharides did not completely remove the secondary crystallisation, the rate of change was decreased, and proved to be directly proportional to the size of the saccharides used; i.e., the larger the saccharide slower the crystallisation. In addition to the effect on secondary crystallisation, blending of PHBV with saccharides resulted in a decrease in melting temperature, and therefore the processing temperature, finally resulting in a material with improved processability and longer life time [288].

With a view of creating a biodegradable material appropriate for food packaging, PHB and PLA blends were extensively studied. PHB acts as a nucleating agent in these blends, improving the mechanical resistance and barrier performance [289]. However, since both biopolymers are brittle, the resulting blends have poor processability. Development of nanocomposites is one of the strategies to improve the processability. For example, a recent report demonstrated coating of PHA/PLA matrix with graphite nanoplatelets or silver nanoparticles for improved Young modulus and strength [290]. The nanocomposite was prepared by milling the polymer blend and nanofillers under gentle shaking, which allowed the blend to remain intact, while the surface softened enough to allow the adhesion of additives. The resulting pellets were homogenously thin coated by nanomaterials and the processability of these nanocomposites was showcased by injection moulding [290].

The success of nanoclays as nanofillers is due to their structure that allows polymer intercalation *via* van der Waals interactions [291]. However, layered silicates of montmorillonite, saponite, and hectorite show opposing polarities with the hydrophobic PHA, leading to the formation of heterogenous mixtures and therefore likely weaker composites. One of the strategies to improve the nanoclays and PHA interaction is chemical modification of PHA. These chemical modifications include introduction of unsaturated bonds, hydroxyl, carboxyl, epoxy groups, chlorination, etc., to yield PHAs with modified physical properties [292].

While a recent report of creating a PHB-lignin copolymer *via* solvent free ring opening polymerisation claims that this material holds great potential for biomedical application [293], it would be beneficial to investigate its potential for packaging as well. The PHB-lignin copolymer shows increased tensile strength, Young’s modulus, and elongation compared to neat PHB, has tuneable antioxidant activity [293], and thus could be interesting material for active packaging. Another example of biocomposites design is P(HBcoHV) processed with a biobased, biodegradable plasticiser acetyltributylcitrate and calcium carbonate as inorganic filler, followed by compositing with sawdust and pea fibres [294]. It was shown that with the increase in the fibre content, the Young’s modulus slightly increased and the elongation at break significantly decreased due to the stiffening effect induced by the lignocellulosic fillers, rendering these biocomposites candidates for single use food packaging [294].

The reports on medium chain length (*mcl*) PHA-based polymers with potential packaging application are scarce. Recently, Rigouin and colleagues reported on a *mcl*PHA homopolymer of (*R*)-3-hydroxydodecanoic acids, with a molar mass (Mw) of 316,000 g/mol, a soft thermoplastic with potential applications in packaging [295]. This homopolymer produced by an engineered yeast *Yarrowia lipolytica* showed high tensile strength and low Young’s modulus, similarly to polyethylene [295]. It would be interesting to test the processability of this polymer.

*Mcl*PHA were used as a coating for bacterial cellulose-based nanopapers and the resulting films showed improved hydrophobicity and transparency compared to cellulose alone [296]. *Mcl*PHA was then mixed with apple extract rich in phenolics to add the antioxidant capacity to the nanopapers. In addition to creating an active packaging by doing so, the resulting films containing the apple extract were much more hydrophobic than the neat nanopapers and the oxygen permeability of the films decreased with the presence of extracts. It is worth mentioning that all materials used to prepare the films were obtained from cider by-products, therefore providing a valorisation route to the agri-waste [296].

## 6. Other Applications

PLA is one of the most popular materials used in 3D printing. 3D printing, or additive manufacturing, allows for the production of a large number of various shapes parts at a competitive cost. The fused deposition modelling (FDM), generally used for low volume production, is a type of 3D printing where a thermoplastic filament is heated above the melting temperature and then extruded onto a print surface in layers. Since PLA has a low melting point, it is attractive material for 3D printing, as it requires less energy for printing compared to other materials, such as acrylonitrile butadiene styrene and polyamides [297]. A very attractive idea is that the use of recycled PLA is a viable option for 3D printing, adding to already positive features of this technology such as savings on raw materials, low energy cost, as well as low CO_2_ emissions [298].

PLA was also tested as a material for the design of composites with halloysite nanotubes, with the aim to achieve the mechanical properties suitable for automotive applications [299]. Tributyl citrate was used as a plasticiser at different concentrations, and it was shown that depending on the plasticiser content, compromises between high tensile and flexural strengths; rigidity and impact resistance; ductility; and crystallisation rate and degree of crystallinity, could be achieved, which is important for automotive applications.

*Mcl*PHAs are used as a source of chiral (*R*)-3-hydroxyalkanoic acids (R3HAs), which are precursors for the synthesis of value-added chemicals including antibiotics, pharmaceuticals, vitamins [300]. (*R*)-3-hydroxydecanoic acid obtained *via* polymer depolymerisation was conjugated to DP18 peptide and its derivatives containing D-amino acids and improved the anti-proliferation activity [31]. The DP18 peptide activity was shown to be dependent on the chain length of R3HAs used for the peptide modification, with (*R*)-3-hydroxynonanoic and (*R*)-3-hydroxydecanoic acid being the most effective at improving DP18 activity [29]. (*R*)-3-hydroxyoctanoic acid also obtained *via mcl*PHA depolymerisation was used as a synthon to generate a library of derivatives, including halogen-, methyl-, benzyl, oxo-derivatives [301]. It was found that the derivatives containing a carboxylic group have low antibacterial and higher antifungal properties, and no in vitro toxicity. In addition, these compounds appear to be affecting bacterial and fungal signalling molecules.

Carboxylated *mcl*PHAs were used for the preparation of stable nanoparticle suspensions using a mixture of ionic and non-ionic surfactants [302]. Dense nanoparticle formulations (>0.4% solids) containing carboxylated *mcl*PHAs could be used to make xerographic toners, hot melt adhesives, and protective coatings, and as microbeads in personal care products such as cosmetics and toothpaste.

## 7. Concluding Remarks and Future Perspectives

If we are to replace single use plastics with bioplastics and avoid further plastic waste accumulation, it is essential to understand what the waste management options are for these materials. The behaviours of different biodegradable plastics in a range of environments have to be assessed, and it must be determined under which conditions plastics show complete biodegradation. The critical factor determining factor for whether a bioplastic will be suitable for a particular waste management option is time. For example, the currently employed international standards (ISO and ASTM) for the biodegradation propose that a material is considered biodegradable in water environments if it shows complete degradation in 56 days, and in soil if it biodegrades in 2 years [1]. A noteworthy characteristic of the currently employed international biodegradation standards is that that the applied temperatures for the ISO and EN standard tests are higher than temperatures in the unmanaged environments that these tests mimic. This needs to be considered when shaping plastic waste policies. In addition, the plastic product design has to be informed by the potential end-of-life option.

When it comes to medical applications of biodegradable polymers, similarly to exploiting the designer space when it comes to packaging applications, identifying the right polymer for the right application is affected by features such as polymer chemistry, polymer performance, polymer processing, and device design. For example, a compromise between hydrophilicity and hydrophobicity can be ideal for drug delivery systems but completely inefficient for implants in some tissues. The right degree of elasticity and the ability to sustain cyclic deformation could be ideal for smooth muscle cell regeneration in cardiac prosthetics but not quite useful in osteo-differentiation. The skilled and creative research that shapes the device design for the right purpose (taking in consideration the tailored polymer properties and modifying them when necessary) could increase the number of successful technologies for both nanomedicine and tissue regeneration using biobased polymers. Biobased polymers, given the enormous variability in their composition, have huge potential for many biomedical applications, but it is the combination of biopolymers that holds the greatest promise for fully unlocking their potential.

## Figures and Tables

**Table 1:** Categorisation of plastic types [40].

	Bio Based Plastics	Oil Based Plastics
**Biodegradable plastics**	Polylactic acid (PLA)	Polycaprolactone (PCL)
	Polyhydroxyalkanoates (PHA)	Polybutylene succinate (PBS)
	Cellulose	Polybutylene adipate (PBA)
	Starch Bio-polybutylene succinate (Bio-PBS)	
**Non-biodegradable plastics**	Bio-polyethylene terephthalate (bio-PET)	Polyethylene terephthalate (PET)
	Bio-polyethylene (bio-PE)	Polyethylene (PE)
	Polyol-polyurethane (P)	Polystyrene (PS) Polypropylene (PP)
